# Into a Shaking Limbo: Case Report of a Nonneoplastic Limbic Encephalitis with Faciobrachial Dystonic Seizures and Parkinsonism

**DOI:** 10.1155/2020/3796807

**Published:** 2020-03-15

**Authors:** Vijay Renga

**Affiliations:** Department of Neurology, Dartmouth Hitchcock Medical Center, Geisel School of Medicine at Dartmouth, One Medical Center Drive, Lebanon, NH 03756, USA

## Abstract

This case report describes a rare but classic presentation of a non-paraneoplastic, antibody-mediated limbic encephalitis. The clinical course did put us in a limbo as it evolved from seizure to Parkinsonism and then from metastasis to stroke, before it finally announced itself by its pathognomonic finding. Knowledge of this rare condition is important as early identification and treatment can change the course.

## 1. Case Scenario

A 63-year-old male was referred to us for an urgent evaluation of new onset left-sided shaking spells for two weeks. He felt that his left side was clenching up suddenly at times. Sometimes he had a choking sensation with short vocalization. Things that he would be holding in his left hand could be thrown away during these spells. By the time he realizes it happening, the spell would be over.

He had a history of renal calculi and experienced hematuria recently. His physician suspected seizures from brain metastases due to possible renal cancer. A CT scan of the head and abdomen was arranged, and the patient was started on carbamazepine. The medicine reduced the frequency of the events but did not stop it. CT head showed pansinusitis without evidence of any metastases. CT abdomen revealed renal stones.

In Neurology clinic, he had few episodes characterized by sudden, involuntary jerking of his left arm. Each of these episodes caught him by surprise. He had Parkinsonism features, more so on the left side with increased tone, bradykinesia, and hyper-reflexia. We were concerned that these spells represented alien limb phenomenon which is typically seen in cortico-basal syndrome. Segmental myoclonus and dystonic spells were also in differentials.

To capture and characterize these spells, he was hospitalized for VEEG monitoring. Multiple spells were captured which had no epileptiform correlates. An MRI of his brain without contrast showed only sinusitis. CSF was unremarkable with 1 cell, protein of 42, and glucose of 88.

**Figure 1:**
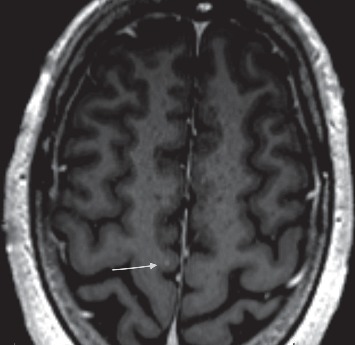
MRI Brain: T1 post-contrast sequence with an arrow pointing to an enhancing lesion over the right motor strip.

A repeat MRI brain with contrast was then obtained. This showed two tiny enhancing lesions, one in the right motor strip (Figure 1) and the other in the left parietal region, suspicious for metastases.

**Figure 2:**
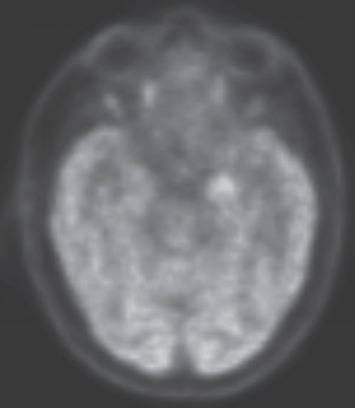
PET scan of brain showing increased activity in the left mesial temporal region.

Outpatient malignancy workup was done with PET scan of the brain and whole body and paraneoplastic panel. PET scan of the body found no primary malignancy. PET scan of the brain showed hyperactivity in the left temporal region, suspicious for epileptic activity (Figure 2). Clinical seizures were on the left side, which did not correlate with this finding. His seizure episodes worsened, and he was started on levetiracetam in addition to increased dose of carbamazepine.

Few days later, he was admitted with generalized rashes from carbamazepine and antiepileptics were changed. He was evaluated by Dermatology, ENT, Oncology, and Gastroenterology for any potential malignancy, and none was detected. We repeated video-EEG monitoring and MRI brain. Video-EEG again captured multiple spells without epileptiform correlate. Seizures became difficult to control with multiple AEDs.

**Figure 3:**
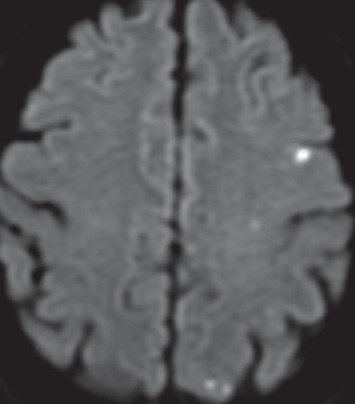
Diffusion MRI sequence showing embolic appearing regions of restricted diffusion (bright white spots) in the left hemisphere.

Repeat MRI showed new scattered embolic infarcts in the left hemisphere (Figure 3) with disappearance of previously seen enhancing lesions that were thought to be metastases. Radiology determined that strokes were possibly misinterpreted as metastases because of the enhancement in the acute phase. Strokes were considered as a potential cause of his seizure at this point. He had extensive stroke workup with vascular imaging, echocardiogram, telemetry, hypercoagulability panel, and vascular doppler, all of which were negative for a cause.

Seizure semiology changed at this point with new right-sided facial spasms and simultaneous arm shaking on the right side, which now correlated with previous PET findings of a left sided process. A diagnosis of paraneoplastic limbic encephalitis was considered. His seizures evolved into the characteristic “Faciobrachial dystonic” appearance previously described in LG1/VGKC antibody-related limbic encephalitis.

Empiric trial of steroids was started and seizures got controlled. He was discharged on oral prednisone and two antiepileptic medications. Few weeks later, his serum paraneoplastic panel came positive for both LG1 and VGKC antibodies while the CSF panel was negative for the same. Caspr2-IgG was negative.

On the clinic follow-up, his seizures had completely abated and he was tapered off steroids. The Parkinsonism features seen during earlier visits also resolved. He continued to have some memory changes.

### 1.1. Final Diagnosis

1. Nonneoplastic limbic encephalitis secondary to VGKC/LG1 antibodies.
2. Embolic strokes of indeterminate etiology

## 2. Discussion

Voltage-gated potassium channel antibodies have been described in nonmalignancy-related limbic encephalitis since the 1990s [1]. Over a decade later, newer antibodies associated with VGKC were characterized. These include (1) leucine-rich glioma protein (LG1) antibodies and (2) contactin-associated protein-like 2 (Caspr2) antibodies. The LGI proteins are secreted by neurons and modulate synaptic excitability. Mutations causing defective LG1 proteins are associated with epilepsy syndromes. Antibodies to LG1 proteins can cause limbic encephalitis. This is the second most common form of antibody-associated limbic encephalitis, after NMDA receptor antibody-related encephalitis [2]. These tend to be clinically less severe than NMDARAb-related encephalitis. Different scenarios can occur with VGKC antibody positive syndromes. These include (1) patients with only VGKC antibody positivity (2) those with VGKC and LG1 positive (as in our patient), and (3) VGKC and Caspr2 positive. Clinical features may give a clue to the underlying antibody type. The VGKC and LG1 positive patients tend to have limbic encephalitis. Memory and behavioral changes are common. Parkinsonism can be seen with striato-limbic encephalitis. While it is not widely reported in association with LG1/VGKC-related encephalitis, this was noted in our patient. A variety of other manifestations including hyponatremia, behavioral disorders, and cognitive issues have been described [3]. Faciobrachial dystonic seizures is the hallmark feature of LG1 antibody-related limbic encephalitis, even though partial and generalized seizures can coexist [4]. Faciobrachial dystonic seizures are rapid and short lasting seizures with facial twitching and involuntary arm jerking. These may not have an epileptic correlate. Semiology is very characteristic and can help diagnose the condition long before the paraneoplastic antibodies are resulted [5]. The lead time can help gain control over the seizure with appropriate treatment.

Caspr2 protein is more widespread and distributed in nerve membranes of peripheral and central nervous systems [3]. Caspr2 antibodies are seen in conditions like Morvan or Isaac's syndrome and associated with peripheral nerve hyperexcitability with fasciculations, muscle spasm, and neuromyotonia along with cognitive changes and seizures [6]. Our patient was Caspr2 negative and did not have any peripheral hyperexcitability related symptoms.

Both these forms of limbic encephalitis respond to steroids or immunotherapy. Relying on the CSF paraneoplastic panel alone could have resulted in a “missed-diagnosis” as serum antibody is more sensitive for LG1 Ab-related limbic encephalitis.

## 3. Conclusion

- VGKC antibody-related limbic encephalitis is commonly a *non-paraneoplastic* limbic encephalitis. In addition to VGKC antibodies, antibodies to LG1 and Caspr2 are now available. Clinical manifestations differ based on the antibody type.
- VGKC with LG1 antibodies are associated with the *pathognomonic faciobrachial dystonic seizures*, which may not have an epileptiform correlate.
- *Striato-limbic encephalitis* with Parkinsonism, while not classically described with VGKC/LG1 antibodies can be seen in this syndrome.
- It is important to test *both serum and CSF paraneoplastic panel antibodies* as either can be false negative. Serum LG1 antibodies are more sensitive than CSF antibodies.
- This condition usually *responds to steroids and immunotherapy.*

## Supplementary Materials

Supplementary Materials A video-EEG recording of a faciobrachial dystonic seizure with sudden jerking movement of the left side. Corresponding EEG shows no epileptiform abnormalities.
